# Proteomics Identifies LUC7L3 as a Prognostic Biomarker for Hepatocellular Carcinoma

**DOI:** 10.3390/cimb46050247

**Published:** 2024-04-27

**Authors:** Yushan Hou, Siqi Wang, Yiming Zhang, Xiaofen Huang, Xiuyuan Zhang, Fuchu He, Chunyan Tian, Aihua Sun

**Affiliations:** 1 State Key Laboratory of Medical Proteomics, National Center for Protein Sciences (Beijing), Beijing Proteome Research Center, Beijing Institute of Lifeomics, Beijing 102206, China; houyushan@ncpsb.org.cn (Y.H.); wangsq0522@163.com (S.W.); zymcell@163.com (Y.Z.); zhangxiuyuanbest@163.com (X.Z.); hefc@nic.bmi.ac.cn (F.H.); 2 College of Life Sciences, Hebei University, Baoding 071002, China; huangxiaofen2001@163.com; 3 Research Unit of Proteomics Dirven Cancer Precision Medicine, Chinese Academy of Medical Sciences, Beijing 102206, China

**Keywords:** hepatocellular carcinoma, alternative splicing, LUC7L3, RRM2, prognostic biomarker

## Abstract

Alternative splicing has been shown to participate in tumor progression, including hepatocellular carcinoma. The poor prognosis of patients with HCC calls for molecular classification and biomarker identification to facilitate precision medicine. We performed ssGSEA analysis to quantify the pathway activity of RNA splicing in three HCC cohorts. Kaplan–Meier and Cox methods were used for survival analysis. GO and GSEA were performed to analyze pathway enrichment. We confirmed that RNA splicing is significantly correlated with prognosis, and identified an alternative splicing-associated protein LUC7L3 as a potential HCC prognostic biomarker. Further bioinformatics analysis revealed that high LUC7L3 expression indicated a more progressive HCC subtype and worse clinical features. Cell proliferation-related pathways were enriched in HCC patients with high LUC7L3 expression. Consistently, we proved that LUC7L3 knockdown could significantly inhibit cell proliferation and suppress the activation of associated signaling pathways in vitro. In this research, the relevance between RNA splicing and HCC patient prognosis was outlined. Our newly identified biomarker LUC7L3 could provide stratification for patient survival and recurrence risk, facilitating early medical intervention before recurrence or disease progression.

## 1. Introduction

Alternative splicing is a ubiquitous regulating mechanism in gene expression that allows the same gene to be transcribed to different messenger RNAs (mRNAs) or non-coding RNAs. This process contributes to cell differentiation, tissue identity acquisition, and organ development [1]. The pre-mRNAs are alternatively spliced by the spliceosome, which consists of small nuclear RNAs (snRNAs) and associated small nuclear ribonucleoprotein particles (snRNPs) and a set of auxiliary proteins. Multiple mechanisms, such as exon skip (ES) and retained intron (RI) are adopted by the spliceosome to accomplish this complex process [2,3]. The alterations in splicing factors and other mis-splicing events detected in tumors suggest that alternative splicing-associated proteins might be potential prognostic biomarkers and targets for tumor patients [4,5,6]. In 2015, Westbrook and his colleagues proposed that spliceosomes are potential therapeutic targets for MYC-driven cancers [7]. In addition, splicing factors PPP1R26 [8], PRPF8 [9], and EIF4A3 [10] have been reported to contribute to the development of HCC through multiple mechanisms.

Liver cancer ranks sixth in terms of incidence and is the fourth most common cause of cancer-related death worldwide [11]. Hepatocellular carcinoma (HCC) accounts for more than 80% of primary liver cancers. Most HCC occurs based on underlying liver diseases, such as chronic hepatitis virus infection (HBV and HCV) or metabolic abnormalities (alcohol abuse, non-alcoholic steatohepatitis (NASH), and non-alcoholic fatty liver disease (NAFLD)). Due to the special anatomical location and physiological function of the liver, many patients have already progressed to advanced HCC at the time of diagnosis and require systemic treatment [12]. The multi-kinase inhibitors sorafenib and lenvatinib are the current first-line therapeutic agents, both of which can prolong the median survival of patients by 2–5 months [13,14,15]. Regretfully, the objective response rates of patients in several clinical trials were less than 30%, suggesting a limited response to current systemic therapeutic agents. Biomarker-driven early diagnosis, molecular staging, and the subsequent precise treatment of HCC patients is an important direction in the development of liver cancer treatment [16]. However, there is no universally recognized prognostic biomarker for HCC yet.

The appliances of multiple omics analysis (genomic, transcriptomic, proteomic, metabolomic, etc.) have become important methods for tumor biomarker discovery [17,18,19]. For proteomics, abnormal alterations in global protein expression patterns suggest important biological processes for tumor progression. Here, we obtained clinical proteomic data from three independently published HCC cohorts and identified LUC7-like 3 pre-mRNA splicing factor as a prognostic biomarker for patients with HCC.

LUC7L3 is a homolog of the yeast U1 snRNA-associated splicing factor Luc7p. In vertebrates, this family consists of three members, LUC7L, LUC7L2, and LUC7L3. LUC7L3 has cysteine/histidine motifs and leucine zipper-like repeats at the N-terminal end. At the same time, arginine and glutamate residues (REs) and arginine and serine residues (RSs) are located at the C-terminus. This protein is enriched at the 5′ end of U1 and U11 snRNAs and binds to the 5′ splicing sites (SSs) of pre-mRNAs [20,21]. LUC7L3 has been reported to play a role in human heart failure through the aberrant splicing of sodium channels [22] and to be involved in HBV replication through the regulation of enhancer II/basal core promoter (ENII/BCP) [23]. The role of LUC7L3 in cancers, especially HCC, has not been elaborated.

In this study, based on previously published proteomic data, we report LUC7L3 as a new biomarker for HCC. Its expression is significantly correlated with patients’ overall survival (OS), disease-free survival (DFS), and clinical features. GO and GSEA analyses demonstrate that LUC7L3 expression is markedly related to the activation of cell proliferation-associated pathways. The knockdown of LUC7L3 significantly inhibited tumor cell growth.

## 2. Materials and Methods

### 2.1. Data Obtaining and Processing

Proteomic data and clinical information were obtained from three published independent HCC patient cohorts: (1) Xing’s cohort (*n* = 152) [24]; (2) Gao’s cohort (*n* = 159) [25]; and (3) Jiang’s cohort (*n* = 101) [26].

To explore the relevance between the RNA splicing pathway and the prognosis of HCC patients, we selected the “GOBP_RNA_SPLICING” gene set from the Molecular Signatures Database (MSigDB) v7.4 and calculated the enrichment score for each sample using the single sample gene set enrichment analysis (ssGSEA) method by the R package GSVA v1.42.0. In addition, we used the R package Survminer v0.4.9 to determine the best cut-off for overall survival and calculate the log-rank *p* values and the hazard ratios (HRs). The survival analysis was performed using a Kaplan–Meier procedure. Hazard ratios (HRs), corresponding 95% confidence intervals (CIs), and the statistical significance of the difference were calculated using the Cox proportional hazards model and Wald test.

Differentially expressed genes between tumor and peritumor tissues were calculated using the R package limma v3.50.3. The associations between LUC7L3 expression and progressive HCC characteristics, including HCC proteomic subtypes, Barcelona Clinic Liver Cancer classification (BCLC), microvascular invasion, differentiation, as well as tumor diameter, were analyzed using the Wilcoxon rank-sum test.

To explore the pathway alteration between different LUC7L3-expressed specimens or cell lines, after calculating the differentially expressed genes (DEGs) between the two groups, GSEA was performed with Molecular Signatures Database (MSigDB) set v7.4, C5: Gene Ontology gene sets (browse 10461 gene sets) using the R package GSVA.

Enrichment analyses of gene ontology (GO) were conducted on the DEGs by using the R package clusterProfiler v4.2.2. A *p* value less than 0.05 was considered significant in all the analyses. The figures were plotted by the ggplot2 R package.

### 2.2. Cell Culture and siRNA Transfection

The human HCC cell lines PLC/PRF/5, Huh7, Hep3B, and HepG2 were purchased from Cell Bank/Stem Cell Bank, Chinese Academy of Sciences. MHCC-97H, MHCC-LM3, and MHCC-LM6 were generous gifts from Dr. Ying Jiang (National Center for Protein Sciences). All the cell lines enrolled in this study were cultured in DMEM (Gibco, Grand Island, NY, USA) supplemented with 10% fetal bovine serum (FBS; Newzerum, Christchurch, New Zealand) and 1% Pen Strep (Gibco, Grand Island, NY, USA) at the conditions of 37 °C and 5% CO_2_. All cell lines were tested to be free of mycoplasma contamination.

The siRNAs targeting LUC7L3 were purchased from GenePharma (Suzhou, China), and the sequences were as follows: siLUC7L3-1 5′-GCAGUUGUUGGAUGAGUUATT-3′, siLUC7L3-2 5′-GCGAUACUUACAGAGCUUATT-3′, siLUC7L3-3 5′-GGGUAGAUGACCAUUUGAUTT-3′. siRNAs were transfected into cells using TurboFect reagent (Thermo Fisher Scientific, Waltham, UA, USA). For a 2 mL 6-well-plate cell culture system, 2 μg siRNA or the negative control (siNC) was incubated with 4 μL TurboFect in 200 μL serum-free DMEM basic for 15 min. The MHCC-97H and PLC/PRF/5 cells ready for transfection were approximately 60% of the density. The culture system was replaced with serum-free DMEM basic, and then the transfection mixture was added drop by drop into the culture system. After 8 h of incubation, the cell culture system was replenished with complete DMEM medium. The siRNA-transfected cells were collected at 36 h after transfection for the Western blotting (WB) analysis and proliferation assay.

### 2.3. CCK8 Cell Proliferation Assay

Thirty-six hours after the siRNA transfection, the cells of each group were collected and inoculated in 96-well plates, respectively (5000 cells/well, 3 replicates), and then cultured in the 37 °C 5% CO_2_ cell incubator. From day 1 to day 5, the cells were incubated with FBS-free DMEM basic containing 10% CCK8 (AQ308-3000T, Aoqing Biotechnology, Beijing, China) for 1.5 h. The absorbance was detected at 450 nm. GraphPad Prism (Version 8.0) was used for data analysis.

### 2.4. Western Blotting

Total proteins were extracted from cells using STD lysis buffer (100 mM Tris, 2% SDS, pH 7.6) supplemented with protease inhibitor (A32955, Thermo Fisher Scientific, Waltham, UA, USA). After quantifying the protein concentration with BCA Protein Assay Kit (23227, Thermo Fisher Scientific, Waltham, UA, USA), the lysate was mixed with 1/9 volume of 1 M dithiothreitol (DTT; R0861, Thermo Fisher Scientific, Waltham, UA, USA) and 1/4 volume of 5× SDS loading buffer (50 mM Tris, 2% SDS, 10% glycerol, 0.005% bromophenol blue, pH 6.8; supplemented with 2% β-mercaptoethanol before using), separately. The lysate was heated at 95 °C for 10 min to obtain a fully prepared WB sample.

Samples containing 15 μg total proteins were separated by 10% SDS-PAGE and transferred to a methanol-activated 0.2 μm polyvinylidene difluoride membrane (PVDF; ISEQ00010, MERK, Darmstadt, Germany). After blocking in 5% skimmed milk for 1 h, the membrane was incubated with anti-LUC7L3 (1:1000; 14504-1-AP, Proteintech, Rosemont, IL, USA) or anti-β-Actin (1:5000; 66009-1-Ig, Proteintech, Rosemont, IL, USA) primary antibody at 4 °C overnight. After washing three times with TBS-T, the corresponding horseradish peroxidase (HRP)-conjugated secondary antibody was added at 1:5000 and incubated for 1 h at room temperature. The target protein was visualized with NcmECL Ultra (P10200, NCM Biotech, Suzhou, China).

### 2.5. Proteomic Profiling of LUC7L3-Knockdown Cells

LUC7L3-knockdown MHCC-97H cells were harvested and washed with cold PBS 3 times. Total protein was extracted by sodium deoxycholate (SDC) lysis buffer which contains 100 mM Tris, 1% SDC (A600150-0250, Shanghai, China), 10 mM Tris(2-carboxyethyl)phosphine hydrochloride (TCEP; HY-W011500, MCE, New Jersey, USA), 40 mM 2-Chloroacetamide (CAA; C0267, Sigma-Aldrich, St. Louis, MO, USA), pH 8.5. BCA protein assay was used to determine the protein concentration. Total protein was digested by trypsin at a 50:1 ratio (*w*/*w*) overnight and then was acidified by FA to a final concentration of 1%. After centrifugation, the acidified supernatant was collected and desalted using a homemade C18 stage-tip. Eluted peptides were evaporated for MS analysis, which was conducted by QE-HF for 120 min. Generated peptide fractions were analyzed with the MaxQuant platform and the reference FASTA files for humans were downloaded from the UniProt database.

## 3. Results

### 3.1. RNA Splicing Is Associated with Poor Survival in HCC

Dysregulated RNA splicing is a molecular feature present in almost all tumors [6]. To investigate the role alternative splicing plays in HCC, we obtained the proteomic data of three independent HCC cohorts. Xing’s cohort classified HCC patients into three subtypes, S-I, S-II, and S-III, characterized by metabolism-related pathway activation, moderate proliferative activation, and high-proliferative metastatic activation, respectively [24]. The S-III was the most aggressive subtype, the patients of which had the worst prognosis. Gao’s cohort subdivided HCC patients in this cohort into three subtypes: S-Mb, S-Me, and S-Pf [25]. The least malignant S-Mb had high levels of metabolism-related proteins, and, therefore, retained most normal liver functions in these three subtypes. S-Pf had multiple activated proliferation-related pathways and was the most aggressive subtype. S-Me had an intermediate expression of metabolic and proliferative proteins. Jiang’s cohort classified HCC patients into three subtypes, and S-III was the most malignant one [26]. Patients in S-III were younger and had shorter overall survival and higher postoperative recurrence rates. They also had more aggressive molecular features, including the activation of transforming growth factor beta (TGF-β) and Rho GTPase. 

**Figure 1:**
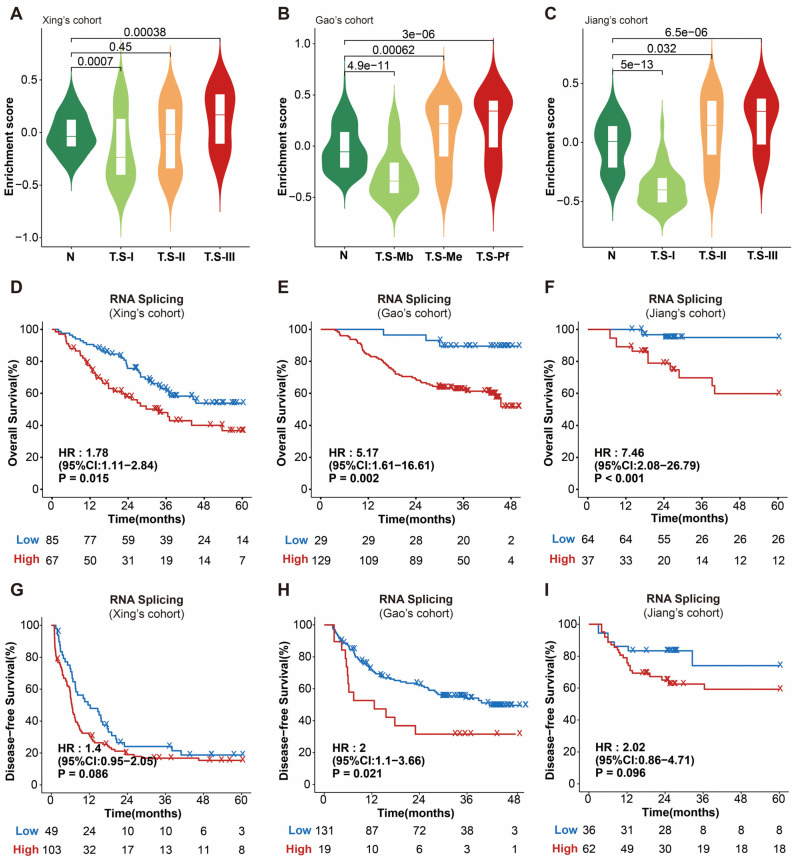
RNA splicing is associated with poor survival in HCC. (**A**–**C**) Violin plot indicating that RNA splicing pathway is significantly enriched in the most malignant HCC subtype. (**D**–**F**) Kaplan–Meier curves indicating that the RNA splicing pathway can stably stratify OS prognosis. (**G**–**I**) Kaplan–Meier curves showing the relevance between RNA splicing pathway and patients’ DFS. HR, hazard ratio; CI, confidence intervals.

We performed ssGSEA analysis on the GOBP_RNA_SPLICING pathway in these cohorts, and the enrichment score was used for further analysis. The results suggested that RNA splicing was significantly elevated in the most malignant subtypes of all three HCC cohorts (Figure 1A–C). Furthermore, the activation of RNA splicing was markedly associated with poor OS prognosis in all three cohorts, with HR values of 1.78 (95% CI: 1.11–2.84, *p* = 0.015), 5.17 (95% CI: 1.61–16.61, *p* = 0.002) and 7.46 (95% CI: 2.08–26.79, *p* < 0.001), respectively (Figure 1D–F). As for DFS, RNA splicing significantly stratified the prognosis of HCC patients in Gao’s cohort, with a noticeable trend toward stratification in the other two cohorts as well (Figure 1G–I).

### 3.2. LUC7L3 Expression Is Overactivated in HCC Tissues and Correlated with Poor Prognosis

**Figure 2:**
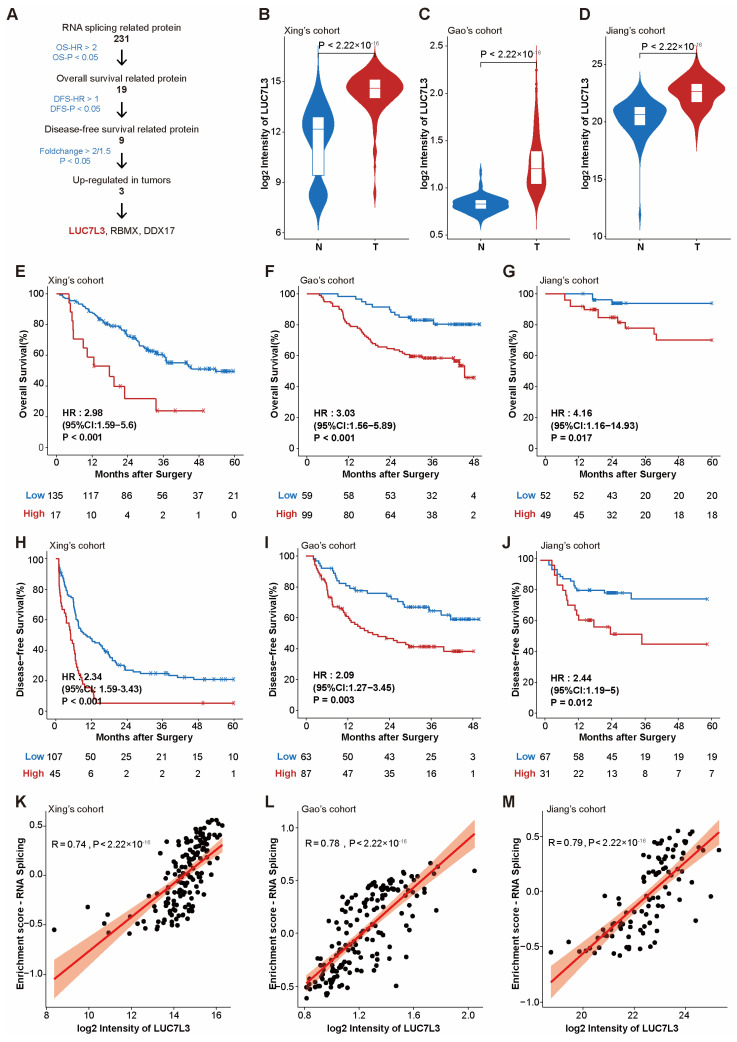
LUC7L3 is a representative alternative splicing-associated protein and significantly correlated with prognosis. (**A**) Workflow scheme of potential HCC biomarker identification. Due to the different proteome quantification techniques, foldchange is required to be greater than 1.5 in Gao’s cohort and greater than 2 in the remaining cohorts. (**B**–**D**) Violin plots of LUC7L3 expression in different subtypes in the three cohorts. (**E**–**G**) Kaplan–Meier survival analysis for OS of HCC patients with different LUC7L3 expression in the three cohorts. (**H**–**J**) Kaplan–Meier survival analysis for DFS of HCC patients with different LUC7L3 expression in the three cohorts. (**K**–**M**) Spearman analysis for the correlation between LUC7L3 expression and RNA splicing pathway. The red line represents the regression line, while the orange shadow shows the 95% confidence interval. OS, overall survival; DFS, disease-free survival.

In the 231 RNA splicing-related proteins identified in the three cohorts, we screened for candidate HCC biomarkers as follows: (1) significantly correlated with poor prognosis with OS (OS-HR > 2, OS-P < 0.05); (2) significantly associated with poor prognosis with DFS (DFS-HR > 1, DFS-P < 0.05); (3) highly expressed in cancer tissues (Foldchange > 2 or 1.5, *p* < 0.05) (Figure 2A). Three proteins, LUC7L3, RNA binding motif protein X-linked (RBMX), and DEAD-box helicase 17 (DDX17), were finally identified as candidate unfavorable biomarkers for HCC. As shown in the Supplement Table S1, LUC7L3 had the highest mean DFS HR among all three cohorts and was most significantly upregulated in tumor tissues. We chose LUC7L3 to perform further investigations.

Compared with that in peritumor tissue, the expression of LUC7L3 was significantly elevated in HCC tumor tissue (Figure 2B–D). This overexpression significantly correlated with shorter overall survival and a higher risk of recurrence in patients (Figure 2E–J), which indicated the potential of LUC7L3 to become a biomarker.

### 3.3. Overexpression of LUC7L3 Positively Correlated with More Progressive Proteomic and Clinical Features

**Figure 3:**
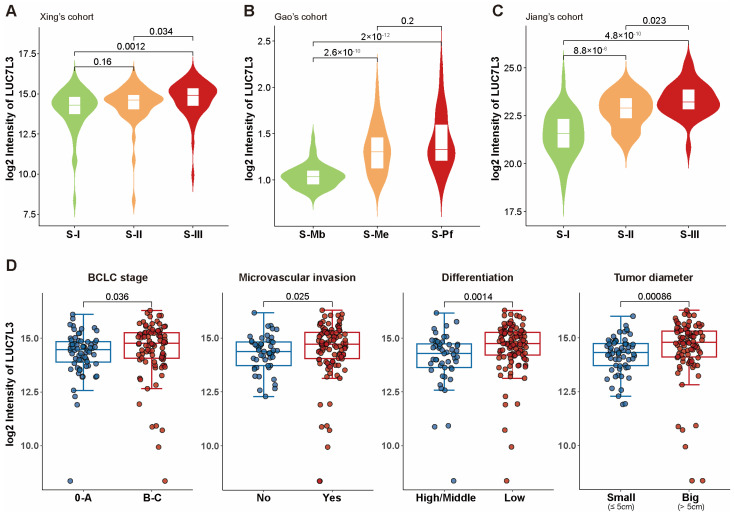
LUC7L3 expression is correlated with aggressive proteomic subtypes and clinical features. (**A**–**C**) Violin plots of LUC7L3 expression among the three subtypes of HCC. (**D**) Box plots depicting the correlation between LUC7L3 expression and the BCLC stage, microvascular invasion, differentiation, and tumor diameter, respectively. BCLC, Barcelona Clinic Liver Cancer.

As aforementioned, patients of the S-III subtype in Xing’s cohort, the S-Pf subtype in Gao’s cohort, and the S-III subtype in Jiang’s cohort had the worst prognosis. We found that LUC7L3 was significantly overexpressed in the most malignant subtype in all three cohorts (Figure 3A–C).

In the clinical, Barcelona Clinic Liver Cancer classification (BCLC) stage, microvascular invasion, differentiation, and tumor diameter were commonly used features to represent the aggressiveness of HCC. We found that patients with higher BCLC stage, positive microvascular invasion, lower differentiation, and larger tumor diameters had significantly higher LUC7L3 expression (Figure 3D). In conclusion, LUC7L3 overexpression indicates a more progressive phenotype of HCC, a worse prognosis of patients, and a higher demand for medical intervention.

### 3.4. Increased LUC7L3 Expression Relates to Activated Cell Proliferation-Related Pathways

**Figure 4:**
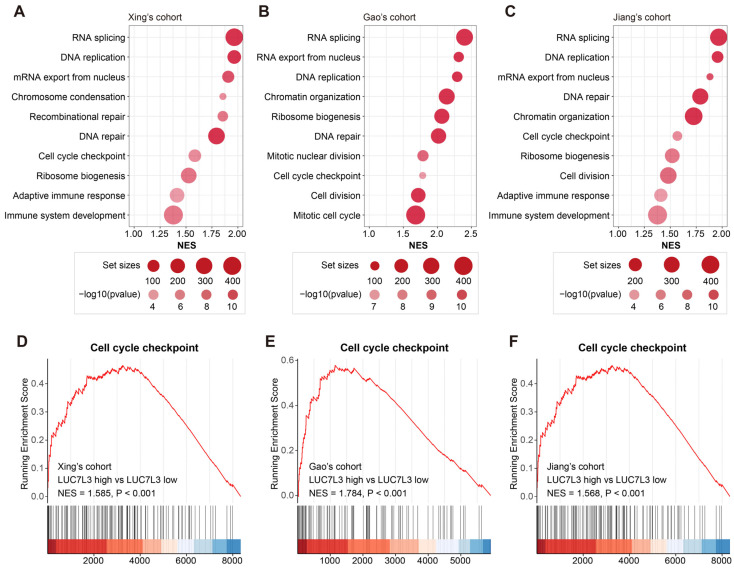
Cell proliferation-related pathways are more active in patients with high expression of LUC7L3. (**A**–**C**) Dot plots indicating the significantly enriched pathways in the LUC7L3 high-expression group in the three HCC cohorts. (**D**–**F**) GSEA of cell cycle checkpoint pathway enrichment in HCC patients with high LUC7L3 expression in the three cohorts. NES, normalized enrichment score.

In all three cohorts, LUC7L3 was highly expressed in the most malignant subtype, which was characterized by progressive cell proliferation (Xing’s cohort S-III, Gao’s cohort S-Pf, and Jiang’s cohort S-III). Therefore, we performed GO enrichment analysis of LUC7L3 in the three cohorts. The results suggested that there was a significant enrichment in cell proliferation-related pathways, including DNA replication, mRNA export from the nucleus, cell cycle checkpoint, and ribosome biosynthesis in the LUC7L3 high-expression group (Figure 4A–C). 

Subsequent GSEA analysis revealed significant activation in the cell cycle checkpoint pathway in the LUC7L3 high-expression group across all three cohorts. The normalized enrichment score (NES) was 1.585 (*p* < 0.001), 1.784 (*p* < 0.001), and 1.568 (*p* < 0.001), respectively (Figure 4D–F).

To sum up, LUC7L3 expression is closely associated with cell proliferation activation.

### 3.5. LUC7L3 Knockdown Inhibits HCC Cell Proliferation

**Figure 5:**
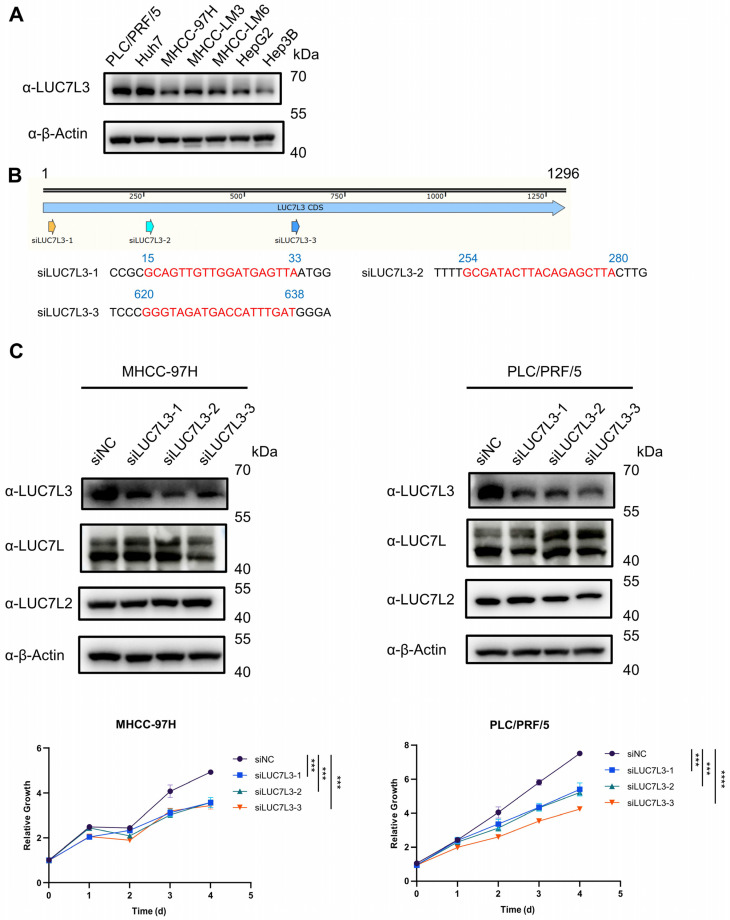
In vitro LUC7L3 knockdown inhibits the proliferation of HCC cells. (**A**) Basic expression of LUC7L3 in HCC cell lines. (**B**) Schematic depiction of the three LUC7L3 siRNAs locus in the LUC7L3 gene. The target sequences have been marked in red. (**C**) HCC cells were transfected with LUC7L3-siRNAs, and cell proliferation was determined with CCK-8 assay. The proliferation assays were carried out in triplicate. NC, negative control. *** *p* < 0.001, **** *p* < 0.0001.

Firstly, we examined the basal expression of LUC7L3 in HCC cell lines. Consistent with the theory that alternative splicing is a fundamental biological process in eukaryotic lives, there was a ubiquitous expression of LUC7L3 in all the detected HCC cell lines. PLC/PRF/5 and Huh7 had high LUC7L3 expression levels, Hep3B had a relatively low expression of LUC7L3, while other cell lines had an intermediate expression level (Figure 5A).

To investigate the effect of LUC7L3 knockdown on HCC cells, we designed and synthesized three LUC7L3 siRNAs (Figure 5B), and transfected them into PLC/PRF/5 and MHCC-97H. The results showed that the proliferation rates of PLC/PRF/5 and MHCC-97H were significantly reduced when LUC7L3 expression was decreased (Figure 5C) without interfering with the expression of LUC7L and LUC7L2.

To further validate the results of clinical proteomic data, we collected LUC7L3-knockdown MHCC-97H cells and the corresponding negative control, extracted total proteins, digested them into peptides, and performed proteomic profiling. 

**Figure 6:**
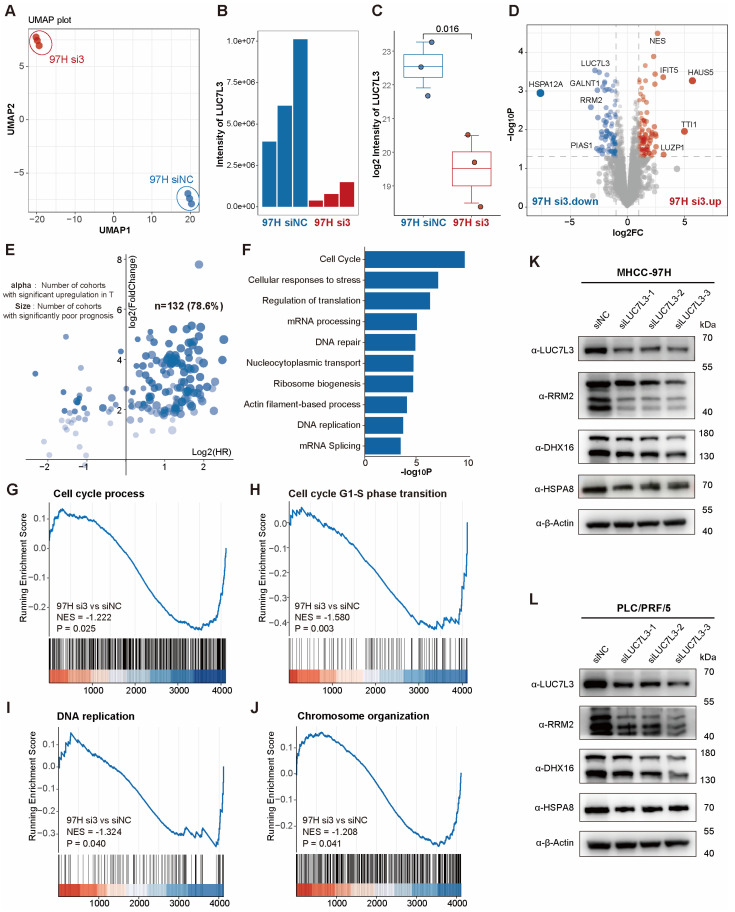
LUC7L3 knockdown significantly down-regulates the proliferation-related pathways of MHCC-97H cells. (**A**) UMAP plot depicting that LUC7L3-knockdown and corresponding control MHCC-97H cells have different protein expression patterns. (**B**,**C**) Column (**B**) and box (**C**) plots indicating that the LUC7L3 level in the knockdown group is significantly down-regulated. (**D**) Volcano plot showing significantly changing genes in the knockdown group. Red dots represents significantly upregulated proteins, and blue dots represents significantly downregulated proteins. The proteins whose expressions have not been significantly altered are showed as gray dots. (**E**) Tumor-related expression characteristics of significantly down-regulated genes in LUC7L3 knockdown group. The X value corresponds to the average OS-HR of the three cohorts, and the Y value corresponds to the ratio of expression abundance in the tumor and adjacent tissue in the three cohorts. Genes positioned in the first quadrant are upregulated during tumorigenesis and are associated with poor prognosis. The transparency of blue dots represents the number of cohorts in which the protein is significantly upregulated in tumor tissue. Deep blue shows that the represented protein is significantly upregulated in all three cohorts. (**F**–**J**) Down-regulated genes are mainly enriched in cell proliferation-related pathways. GO analysis the down-regulated proteins in LUC7L3 knockdown cells (**F**), GSEA of cell cycle process (**G**), Cell cycle G1-s phase transition (**H**), DNA replication (**I**) and Chromosome organization (**J**) in LUC7L3 knockdown cells. (**K**,**L**) Western Blot depicting that the expression of RRM2, DXH16, and HSPA8 are down-regulated after LUC7L3 knockdown, which is consistent with the proteomic profiling. UMAP, Uniform Manifold Approximation and Projection; NC, negative control; NES, normalized enrichment score.

Uniform manifold approximation and the projection (UMAP) plot suggested that the LUC7L3-knockdown and control group had different protein expression patterns (Figure 6A). As detected by mass spectrometry, there was a significant reduction in LUC7L3 protein abundance in the knockdown group (Figure 6B,C) and a common down-regulation in cell cycle-associated proteins such as RRM2 (Figure 6D). We further explored these co-down-regulated proteins and found that approximately 80% of them were overexpressed in tumor tissues and correlated with poor prognosis in HCC cohorts (Figure 6E).

Subsequently, we performed GO enrichment analysis on the LUC7L3 knockdown group and found that, consistent with the data in the clinical samples, the knockdown of LUC7L3 resulted in the inhibition of the cell cycle, DNA replication, ribosome biosynthesis, and other cell proliferation-related pathways (Figure 6F). GSEA analysis also showed that cell cycle process (NES = −1.222, *p* = 0.025), DNA replication (NES = −1.324, *p* = 0.040), and chromosome organization (NES = −1.208, *p* = 0.041) were significantly down-regulated after the LUC7L3 knockdown (Figure 6G–J).

### 3.6. LUC7L3 Co-Down-Regulated Protein RRM2 Is Correlated with Prognosis in HCC and Can Serve as a Potential Therapeutic Target

One of the co-down-regulated proteins identified in the LUC7L3-knockdown group was ribonucleotide reductase subunit M2 (RRM2), which had been reported to contribute to the progression of multiple cancers [27,28] and serve as a potential therapeutic target. More specifically, several studies have suggested that the overexpression of RRM2 was involved in the development of acquired resistance to chemotherapeutic agents such as gemcitabine, temozolomide, and so on [29,30,31].

**Figure 7:**
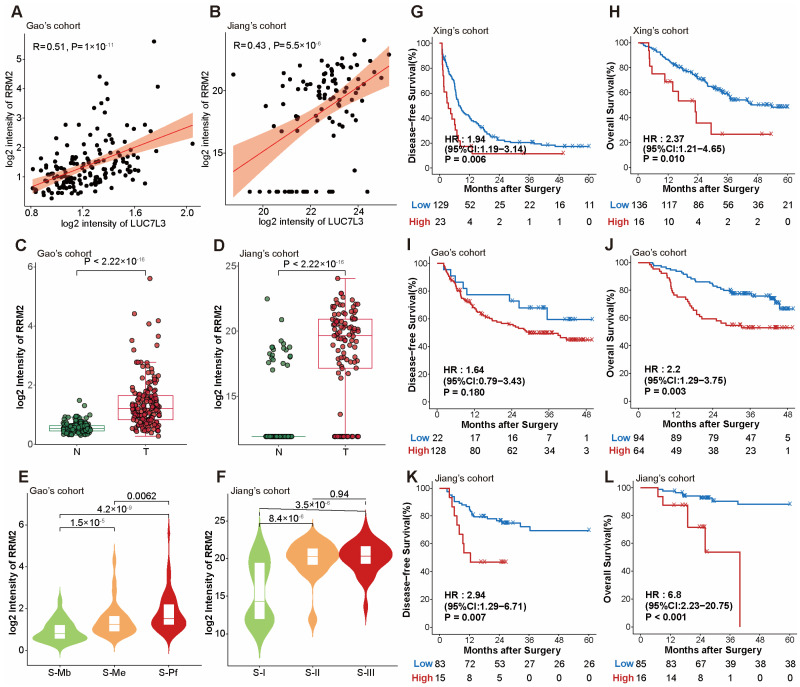
RRM2 expression is altered in HCC and correlates to patients’ outcomes. (**A**,**B**) Spearman analyses showing that LUC7L3 and RRM2 are co-expressed in Gao’s cohort and Jiang’s cohort. The red line represents the regression line, while the orange shadow shows the 95% confidence interval. (**C**,**D**) Box plots indicating that RRM2 is overexpressed in tumor tissues. (**E**,**F**) Violin plots depicting that RRM2 expression level is elevated in more malignant HCC subtypes. (**G**–**L**) Kaplan–Meier curves indicating that RRM2 expression could stratify the survival of HCC patients in the three cohorts. N, non-tumor tissue; T, tumor tissue.

We explored the expression pattern of RRM2 in the HCC cohorts. The results suggested that RRM2 was co-expressed with LUC7L3 in HCC patients, which was consistent with the in vitro knockdown assay (Figure 7A,B). RRM2 exhibited elevated expression in tumor tissue, which was amplified with an increased malignancy of the HCC subtype (Figure 7C–F). Survival analysis demonstrated a significant correlation between RRM2 expression and both OS and DFS across all three cohorts, despite the relatively low abundance of RRM2 detected in Xing’s cohort. Given the observed correlation between RRM2 and LUC7L3 in both clinical cohorts and the in vitro assay, alongside RRM2’s reported potential as a target for overcoming resistance to chemotherapeutic agents, we speculated that LUC7L3 might similarly serve as a target for therapeutic intervention.

## 4. Discussion

As a major global health concern, research on HCC has received extensive attention. Due to unique physiological functions and genetic characteristics, HCC patients are often diagnosed in their advanced stages. Multikinase inhibitors such as sorafenib and lenvatinib remain the first-line treatment for advanced HCC currently, which prolongs the survival of patients by approximately 2–3 months [13,15]. In 2020, a phase III trial of the immune checkpoint inhibitor Atezolizumab with the vascular endothelial growth factor (VEGF) monoclonal antibody Bevacizumab in patients with unresectable HCC was conducted [32]. The combination regimen significantly prolonged OS and DFS compared to sorafenib, advancing the development of immunotherapy in HCC treatment. However, the objective responsive rates of both Atezolizumab plus Bevacizumab and sorafenib in HCC patients were less than 30%, suggesting that patients had limited response to current systemic therapies. Biomarker-guided molecular classification and precise medicine administration based on patients’ subtypes is the direction of the development of tumor treatment, including HCC. In Jiang’s cohort, researchers identified sterol O-acyltransferase 1 (SOAT1) as a biomarker for the S-III subtype with the highest malignancy, and its high expression was significantly associated with the poor prognosis of patients [26]. Furthermore, Jiang and her co-workers suggested that SOAT1 can be a potential therapeutic target for patients in the S-III subtype. In Gao’s cohort, researchers identified pyrroline-5-carboxylate reductase 2 (PYCR2) and alcohol dehydrogenase 1A (ADH1A) as biomarkers of poor and good prognosis in HCC patients, respectively [25]. Therefore, it is convincing that deep analysis of proteomic data will provide us with a more comprehensive understanding of the molecular signature of HCC and contribute to the identification of potential biomarkers and targets.

Alternative splicing is a ubiquitous physiological process in eukaryotic organisms. The role of alternative splicing in the control of cell proliferation and cancer has been extensively studied for decades. In the context of non-cancerous liver regeneration, the alternative splicing pattern is globally altered. The down-regulation of the splicing regulator epithelial splicing regulatory protein 2 (ESPR2) promotes the activation of the Hippo signaling pathway in neonatal hepatocytes, which, in turn, facilitates liver regeneration [33]. Whereas in cancer, the activation of splicing factor expression [34], mutations [35], or post-translational modifications [36] is also closely associated with the progressive proliferation of cancer.

To explore the role of alternative splicing in HCC, we performed ssGSEA analysis in Xing’s cohort, Gao’s cohort, and Jiang’s cohort and found that the RNA splicing pathway enrichment score could differentiate the prognosis of patients’ overall survival. The activation of the RNA splicing pathway was significantly associated with poor prognosis. Through Cox regression analysis, we found that multiple alternative splicing-related proteins were associated with a worse prognosis of HCC patients. We identified LUC7L3 as a potential HCC biomarker, as its expression in tumor tissue was markedly elevated, and significantly correlated with worse OS and DFS prognoses.

As a candidate biomarker, LUC7L3 expression was significantly activated in the most progressive subtype among the three cohorts. Meanwhile, a high level of LUC7L3 was significantly correlated with higher BCLC stage, microvascular invasion existence, and poor differentiation, which further strengthened the significance of LUC7L3 expression as an indicator of HCC malignancy. In addition, we compared the ability of LUC7L3 with the existing liver cancer biomarkers AFP and DCP to stratify HCC patients (Figure S1B), showing that they had similar abilities in predicting the prognosis of patients with full-stage HCC (Gao’s cohort and Xing’s cohort). Moreover, as a representative protein of the LUC7L family, LUC7L2 has a similar prognostic stratification ability to AFP and DCP in patients with full-stage HCC. However, for patients with early-stage HCC (Jiang’s cohort), the prognostic stratification ability of LUC7L3 is much better than that of AFP, indicating that LUC7L3 is beneficial for the prognostic diagnosis of HCC patients, especially for early-stage HCC. As with most under-investigation biomarkers, MS may not be suitable for clinical diagnosis. Consequently, there is a necessity to develop clinical testing-appropriate methods.

To further investigate the role of LUC7L3 in HCC, we performed GO and GSEA enrichment analyses in the three cohorts. Consistent with previous reports, LUC7L3, as an alternative splicing-related protein, mainly participated in pathways associated with cell proliferation, such as DNA replication, RNA splicing, ribosomal biosynthesis, cell cycle process, and so on.

Progressive proliferation is one of the hallmarks of cancer [37]. The cell cycle is a series of tightly regulated events leading to the faithful replication of the cell genome and the generation of two daughter cells. The cell cycle can be divided into the following four main phases: gap1 (G1), in which the metabolism, RNA transcription, and ribosome biogenesis are activated to prepare the material and energy required by subsequent phases [38]; DNA synthesis (S); gap2 (G2), where the DNA replicate is terminated and a large amount of mitosis-associated proteins are expressed; and mitosis (M) [39,40]. Cyclins and their catalytic partners, cell cycle protein-dependent kinases (CDKs), regulate the progression from one cell cycle phase to another. As aberrant growth signals in cancer cells often result in the presence of the overactivation of cell cycle-associated proteins, targeting the cell cycle appears to be an effective way to inhibit tumor growth [41]. The alternative splicing of pre-mRNAs exists in several cell cycle-related proteins, such as CDK2 and CDC-like kinase 1 (CLK1), which further affects the cell cycle process by influencing their kinase activity [42]. Furthermore, alternative splicing could also alternatively affect the cell cycle process by regulating other signaling pathways [43,44].

The cell cycle checkpoint pathway was also significantly enriched in the LUC7L3 high-expression group of HCC patients. Cell cycle checkpoints are essential for cell cycle ordering and genome homeostasis during cell proliferation. According to the specific function, they can be categorized into DNA damage checkpoints and DNA replication stress checkpoints [45]. Of these, the DNA damage checkpoint (ATM-CHK2-p53) primarily monitors genetic errors and blocks cell cycle progression to promote DNA repair. Unfortunately, mutations in DNA damage checkpoint-related genes are common in malignant tumors [46].

To validate the results obtained from clinical proteomic data, we used siRNA to knock down the expression of LUC7L3 in hepatocellular carcinoma cell lines and found that cell proliferation was significantly inhibited. The proteomic profiling of LUC7L3-knockdown MHCC-97H and the corresponding negative control also demonstrated that the genetic knockdown of LUC7L3 expression led to a significant down-regulation of cell proliferation-related pathways.

RRM2 is one of the co-down-regulated proteins in LUC7L3-knockdown MHCC-97H cells, which has been extensively studied and suggested to be a potential therapeutic target. Two RRM2 combine with two larger subunits RRM1 to form a protein tetramer ribonucleotide reductase (RR), which is responsible for catalyzing the reaction of nucleotides to deoxynucleotides, generating DNA synthesis precursors dNTPs [47]. Multiple research works have reported that RRM2 could contribute to chemotherapy resistance. One mechanism is that camptothecin-induced DNA damage promotes RRM2 deacetylation by enhancing Sirt2-RRM2 interaction, while the acetylation of RRM2 could disrupt RR assembly, which provides tumor cells with abundant dNTPs for DNA repair and survival [48]. There are various strategies available for RRM2-targeting therapeutic agents, including destroying the free radical and iron center with antioxidants, decreasing RRM2 mRNA level with oligonucleotides, and directly inhibiting the active sites with small molecule inhibitors [49]. RRM2 facilitates DNA replication and repair by supplying dNTPs through participating in the RR catalytical process, further promoting tumor cell proliferation. On the other hand, LUC7L3 contributes to cell proliferation by engaging in alternative splicing. The correlation in their functions implies that LUC7L3 could hold promise as a potential drug target.

## 5. Conclusions

In summary, we report the potential of the alternative splicing-associated protein LUC7L3 as a prognostic biomarker for HCC. We suggest that the overexpression of this protein in HCC tumor tissue might be closely related to the aggressive proliferation of HCC and that patients with high LUC7L3 expression require positive medical interventions as they tend to have shorter OS and possess a higher risk of postoperative recurrence. The molecular mechanism of LUC7L3 promoting tumor cell proliferation, the relationship between LUC7L3 and RRM2, and its potential diagnostic and therapeutic value need to be further clarified.

## Data Availability

The original contributions presented in the study are included in the article, further inquiries can be directed to the corresponding authors.

## Supplementary Materials

The following supporting information can be downloaded at: https://www.mdpi.com/article/10.3390/cimb46050247/s1, Figure S1: LUC7L and LUC7L2 in the clinical proteomic data; Figure S2: Correlation analysis between LUC7L3 and recognized alternative splicing-related proteins, including HSPA8, DHX16, PPIG and SNRNP40; Table S1: Cox analysis and expression level of LUC7L3, RBMX, and DDX17 in proteomic data.
