# Supplementary material for: Proteomics Identifies LUC7L3 as a Prognostic Biomarker for Hepatocellular Carcinoma
Source: Curr Issues Mol Biol. 2024 Apr 27;46(5):4004–20. doi: 10.3390/cimb46050247 (PMC11120364; doi:10.3390/cimb46050247)
Supplement: Supplementary file 1 [file cimb-46-00247-s001.zip › cimb-2944112-supplementary.pdf]

**Table S1.** Cox analysis and expression level of LUC7L3, RBMX, and DDX17 in proteomic data.

**DFS**

| | | | Jiang's cohort | | Gao's cohort | | Xing's cohort | |
|---|---|---|---|---|---|---|---|---|
| **GeneName** | **UniprotID** | **DFS.HR.average** | **HR** | **Pvalue** | **HR** | **Pvalue** | **HR** | **Pvalue** |
| LUC7L3 | O95232 | 2.29 | 2.44 (1.19-5) | 0.012 | 2.09 (1.27-3.45) | 0.003 | 2.34 (1.59-3.43) | <0.001 |
| RBMX | P38159 | 1.96 | 2.3 (1.08-4.92) | 0.027 | 1.79 (1.1-2.91) | 0.018 | 1.8 (1.26-2.57) | 0.001 |
| DDX17 | Q92841 | 1.95 | 2.44 (1.17-5.07) | 0.014 | 1.82 (1.11-2.98) | 0.016 | 1.6 (1.09-2.33) | 0.015 |

**OS**

| | | | Jiang's cohort | | Gao's cohort | | Xing's cohort | |
|---|---|---|---|---|---|---|---|---|
| **GeneName** | **UniprotID** | **OS.HR.average** | **HR** | **Pvalue** | **HR** | **Pvalue** | **HR** | **Pvalue** |
| LUC7L3 | O95232 | 3.39 | 4.16 (1.16-14.93) | 0.017 | 3.03 (1.56-5.89) | 0.001 | 2.98 (1.59-5.6) | <0.001 |
| RBMX | P38159 | 3.10 | 4.59 (1.61-13.14) | 0.002 | 2.34 (1.31-4.16) | 0.003 | 2.38 (1.44-3.91) | <0.001 |
| DDX17 | Q92841 | 3.73 | 5.73 (2.01-16.37) | <0.001 | 2.98 (1.57-5.65) | <0.001 | 2.47 (1.51-4.03) | <0.001 |

**T/P**

| | | | Jiang's cohort | | Gao's cohort | | Xing's cohort | |
|---|---|---|---|---|---|---|---|---|
| **GeneName** | **UniprotID** | **logFC.average** | **logFC** | **Padj** | **logFC** | **Padj** | **logFC** | **Padj** |
| LUC7L3 | O95232 | 1.81 | 2.111 | <0.001 | 0.612 | <0.001 | 2.706 | <0.001 |
| RBMX | P38159 | 1.25 | 1.658 | <0.001 | 0.605 | <0.001 | 1.475 | <0.001 |
| DDX17 | Q92841 | 1.10 | 1.674 | <0.001 | 0.604 | <0.001 | 1.028 | <0.001 |

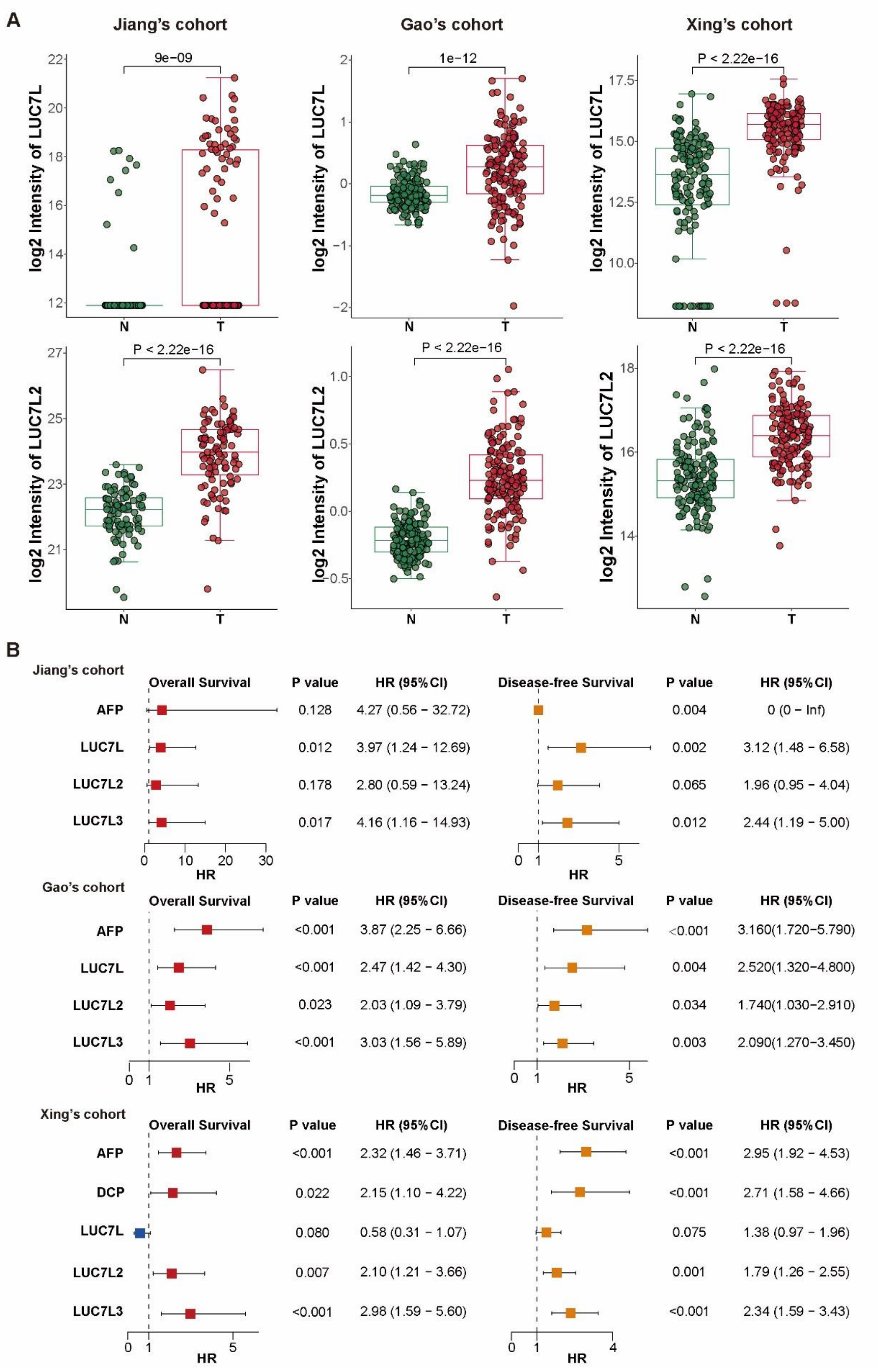


**Figure S1. LUC7L and LUC7L2 in the clinical proteomic data.** (A) Box plots indicating that the expression of LUC7L and LUC7L2 are upregulated in HCC tumor tissue in all three clinical cohorts. (B) Forest plots depicting the Cox analysis of AFP, DCP, LUC7L, LUC7L2, and LUC7L3. AFP, alpha-fetoprotein; DCP, des-γ-carboxy prothrombin.

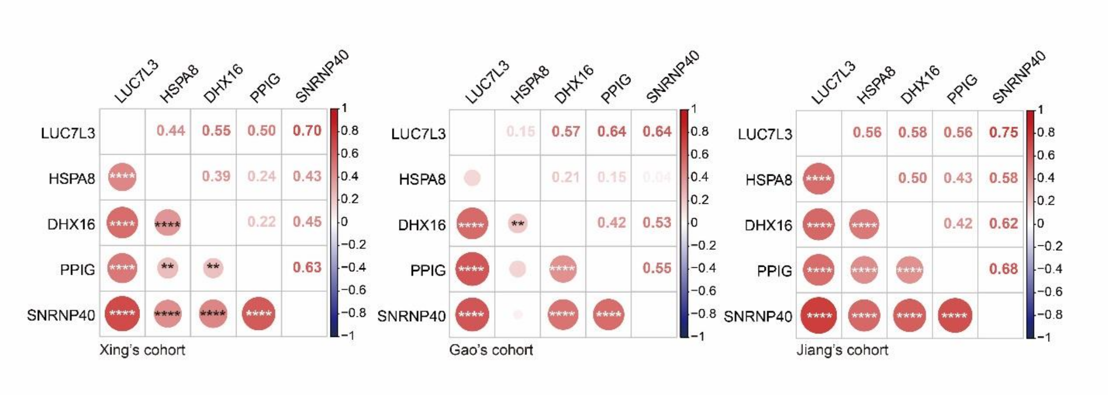


**Figure S2.** Correlation analysis between LUC7L3 and recognized alternative splicing-related proteins, including HSPA8, DHX16, PPIG and SNRNP40.
